# 4-Bromo-2-[(*E*)-(4-fluoro­phen­yl)imino­meth­yl]phenol

**DOI:** 10.1107/S1600536811004429

**Published:** 2011-02-12

**Authors:** Amir Adabi Ardakani, Reza Kia, Hadi Kargar, Muhammad Nawaz Tahir

**Affiliations:** a Islamic Azad University, Ardakan Branch, Iran; b X-ray Crystallography Laboratory, Plasma Physics Research Center, Science and Research Branch, Islamic Azad University, Tehran, Iran; c Chemistry Department, Payame Noor University, Tehran 19395-4697, I. R. of Iran; d Department of Physics, University of Sargodha, Punjab, Pakistan

## Abstract

In the title compound, C_13_H_9_BrFNO, the dihedral angle between the substituted benzene rings is 9.00 (11)°. Strong intra­molecular O—H⋯N hydrogen bonds generate *S*(6) ring motifs.

## Related literature

For standard bond lengths, see: Allen *et al.* (1987 ▶). For hydrogen-bond motifs, see: Bernstein *et al.* (1995 ▶).
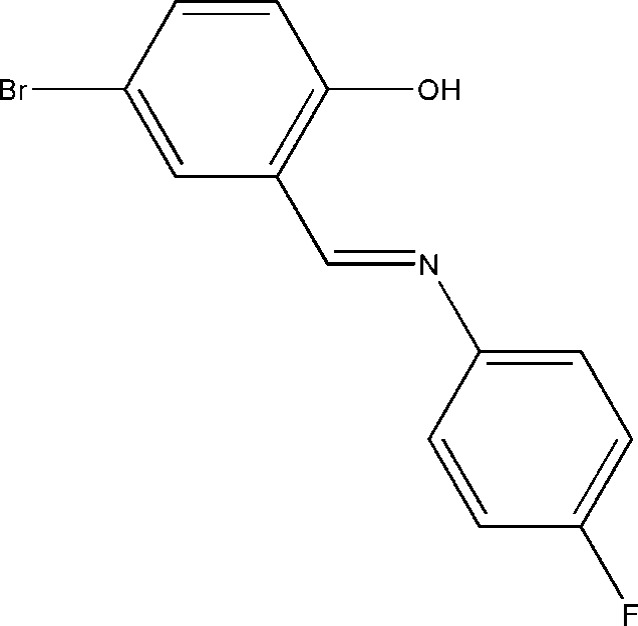


## Experimental

### 

#### Crystal data

C_13_H_9_BrFNO
*M* *_r_* = 294.12
Monoclinic, 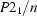

*a* = 4.4820 (2) Å
*b* = 20.8088 (9) Å
*c* = 12.2189 (5) Å
β = 94.570 (2)°
*V* = 1135.97 (8) Å^3^
*Z* = 4
Mo *K*α radiation
μ = 3.61 mm^−1^
*T* = 296 K
0.35 × 0.17 × 0.11 mm

#### Data collection

Bruker SMART APEXII CCD area-detector diffractometer
Absorption correction: multi-scan (*SADABS*; Bruker, 2005 ▶) *T*_min_ = 0.365, *T*_max_ = 0.692
10561 measured reflections
2792 independent reflections
1958 reflections with *I* > 2σ(*I*)
*R*_int_ = 0.034

#### Refinement

*R*[*F* ^2^ > 2σ(*F* ^2^)] = 0.032
*wR*(*F* ^2^) = 0.083
*S* = 1.02
2792 reflections
154 parameters
H-atom parameters constrained
Δρ_max_ = 0.44 e Å^−3^
Δρ_min_ = −0.26 e Å^−3^

### 

Data collection: *APEX2* (Bruker, 2005 ▶); cell refinement: *SAINT* (Bruker, 2005 ▶); data reduction: *SAINT*; program(s) used to solve structure: *SHELXTL* (Sheldrick, 2008 ▶); program(s) used to refine structure: *SHELXTL*; molecular graphics: *SHELXTL*; software used to prepare material for publication: *SHELXTL* and *PLATON* (Spek, 2009 ▶).

## Supplementary Material

Crystal structure: contains datablocks global, I. DOI: 10.1107/S1600536811004429/jh2266sup1.cif

Structure factors: contains datablocks I. DOI: 10.1107/S1600536811004429/jh2266Isup2.hkl

Additional supplementary materials:  crystallographic information; 3D view; checkCIF report

## Figures and Tables

**Table 1:** Hydrogen-bond geometry (Å, °)

*D*—H⋯*A*	*D*—H	H⋯*A*	*D*⋯*A*	*D*—H⋯*A*
O1—H1⋯N1	0.82	1.89	2.612 (2)	146

**Fig. 1.**
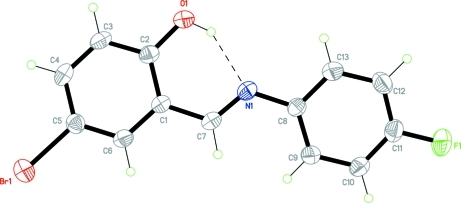
The asymmetric unit of the title compound, showing 40% probability displacement ellipsoids and the atomic numbering. Intramolecular hydrogen bond is drawn as dashed lines.

**Fig. 2.**
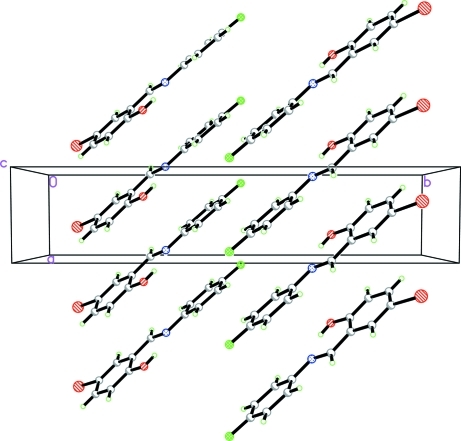
The packing diagram of the title compound, viewed down the c-axis.

## supplementary crystallographic information

### Comment

Schiff base ligands are one of the most prevalent systems in coordination chemistry. As part of a general study of Schiff bases, we have determined the crystal structure of the title compound.

The asymmetric unit of the title compound, Fig. 1, comprises a potentially bidenate Schiff base ligand. The bond lengths (Allen *et al.,* 1987) and angles are within the normal ranges. The dihedral angle between the substituted benzene rings is 9.00 (11)Å. Strong intramolecular O—H···N hydrogen bonds generate *S(6)* ring motifs (Bernstein *et al.*, 1995).

### Experimental

The title compound was synthesized by adding 5-bromo-salicylaldehyde (2 mmol) to a solution of *p*-fluoroaniline (2 mmol) in ethanol (20 ml). The mixture was refluxed with stirring for half an hour. The resulting light-yellow solution was filtered. Pale-yellow single crystals suitable for *X*-ray diffraction were recrystallized from ethanol by slow evaporation of the solvents at room temperature over several days.

### Refinement

H atoms of the hydroxy groups were located by a rotating model and constrained to refine with the parent atoms with U_iso_(H) = 1.5 U_eq_(O), see Table 1. The remaining H atoms were positioned geometrically with C—H = 0.93 Å and included in a riding model approximation with U_iso_ (H) = 1.2 U_eq_ (C).

### Crystal data

**Table:**

C_13_H_9_BrFNO	*F*(000) = 584
*M**_r_* = 294.12	*D*_x_ = 1.720 Mg m^−^^3^
Monoclinic, *P*2_1_/*n*	Mo *K*α radiation, λ = 0.71073 Å
Hall symbol: -P 2yn	Cell parameters from 2520 reflections
*a* = 4.4820 (2) Å	θ = 2.5–28.5°
*b* = 20.8088 (9) Å	µ = 3.61 mm^−^^1^
*c* = 12.2189 (5) Å	*T* = 296 K
β = 94.570 (2)°	Prism, pale-yellow
*V* = 1135.97 (8) Å^3^	0.35 × 0.17 × 0.11 mm
*Z* = 4	

### Data collection

**Table:**

Bruker SMART APEXII CCD area-detector diffractometer	2792 independent reflections
Radiation source: fine-focus sealed tube	1958 reflections with *I* > 2σ(*I*)
graphite	*R*_int_ = 0.034
φ and ω scans	θ_max_ = 28.3°, θ_min_ = 1.9°
Absorption correction: multi-scan (*SADABS*; Bruker, 2005)	*h* = −5→5
*T*_min_ = 0.365, *T*_max_ = 0.692	*k* = −27→27
10561 measured reflections	*l* = −16→16

### Refinement

**Table:**

Refinement on *F*^2^	Primary atom site location: structure-invariant direct methods
Least-squares matrix: full	Secondary atom site location: difference Fourier map
*R*[*F*^2^ > 2σ(*F*^2^)] = 0.032	Hydrogen site location: inferred from neighbouring sites
*wR*(*F*^2^) = 0.083	H-atom parameters constrained
*S* = 1.02	*w* = 1/[σ^2^(*F*_o_^2^) + (0.0413*P*)^2^ + 0.1115*P*] where *P* = (*F*_o_^2^ + 2*F*_c_^2^)/3
2792 reflections	(Δ/σ)_max_ = 0.001
154 parameters	Δρ_max_ = 0.44 e Å^−^^3^
0 restraints	Δρ_min_ = −0.26 e Å^−^^3^

### Special details

**Table:**

Geometry. All esds (except the esd in the dihedral angle between two l.s. planes) are estimated using the full covariance matrix. The cell esds are taken into account individually in the estimation of esds in distances, angles and torsion angles; correlations between esds in cell parameters are only used when they are defined by crystal symmetry. An approximate (isotropic) treatment of cell esds is used for estimating esds involving l.s. planes.
Refinement. Refinement of F^2^ against ALL reflections. The weighted R-factor wR and goodness of fit S are based on F^2^, conventional R-factors R are based on F, with F set to zero for negative F^2^. The threshold expression of F^2^ > 2sigma(F^2^) is used only for calculating R-factors(gt) etc. and is not relevant to the choice of reflections for refinement. R-factors based on F^2^ are statistically about twice as large as those based on F, and R- factors based on ALL data will be even larger.

### Fractional atomic coordinates and isotropic or equivalent isotropic displacement parameters (Å^2^)

**Table:**

	*x*	*y*	*z*	*U*_iso_*/*U*_eq_	
Br1	1.14491 (6)	0.420695 (13)	0.55664 (2)	0.06007 (13)	
F1	−0.3896 (4)	−0.01392 (7)	0.62967 (14)	0.0714 (5)	
O1	0.7822 (4)	0.23124 (8)	0.89264 (12)	0.0502 (4)	
H1	0.6582	0.2057	0.8661	0.075*	
N1	0.4202 (4)	0.17993 (8)	0.73875 (14)	0.0357 (4)	
C1	0.7310 (5)	0.26970 (10)	0.70681 (16)	0.0322 (5)	
C2	0.8605 (5)	0.27266 (11)	0.81485 (16)	0.0356 (5)	
C3	1.0742 (5)	0.31885 (11)	0.84342 (17)	0.0415 (5)	
H3	1.1617	0.3204	0.9150	0.050*	
C4	1.1590 (5)	0.36250 (11)	0.76728 (18)	0.0418 (5)	
H4	1.3019	0.3936	0.7873	0.050*	
C5	1.0300 (5)	0.35970 (10)	0.66087 (18)	0.0377 (5)	
C6	0.8198 (5)	0.31402 (10)	0.63019 (17)	0.0369 (5)	
H6	0.7360	0.3126	0.5581	0.044*	
C7	0.5052 (4)	0.22206 (10)	0.67230 (17)	0.0343 (5)	
H7	0.4212	0.2224	0.6002	0.041*	
C8	0.2060 (4)	0.13210 (10)	0.70442 (17)	0.0332 (5)	
C9	0.1021 (5)	0.12019 (11)	0.59655 (18)	0.0418 (5)	
H9	0.1690	0.1454	0.5406	0.050*	
C10	−0.0999 (5)	0.07125 (11)	0.57144 (19)	0.0460 (6)	
H10	−0.1702	0.0633	0.4990	0.055*	
C11	−0.1946 (5)	0.03478 (11)	0.6549 (2)	0.0445 (6)	
C12	−0.0996 (5)	0.04499 (12)	0.7619 (2)	0.0483 (6)	
H12	−0.1684	0.0195	0.8171	0.058*	
C13	0.1021 (5)	0.09426 (12)	0.78670 (19)	0.0443 (6)	
H13	0.1687	0.1021	0.8595	0.053*	

### Atomic displacement parameters (Å^2^)

**Table:**

	*U*^11^	*U*^22^	*U*^33^	*U*^12^	*U*^13^	*U*^23^
Br1	0.0733 (2)	0.05159 (18)	0.05448 (18)	−0.01654 (13)	−0.00037 (13)	0.01097 (12)
F1	0.0782 (11)	0.0552 (10)	0.0788 (11)	−0.0331 (9)	−0.0069 (9)	0.0023 (8)
O1	0.0592 (10)	0.0587 (11)	0.0313 (8)	−0.0167 (8)	−0.0051 (7)	0.0033 (8)
N1	0.0310 (10)	0.0401 (11)	0.0354 (9)	0.0009 (8)	−0.0014 (7)	−0.0038 (8)
C1	0.0303 (11)	0.0348 (11)	0.0312 (10)	0.0019 (9)	0.0005 (8)	−0.0036 (9)
C2	0.0348 (12)	0.0405 (12)	0.0315 (11)	0.0018 (9)	0.0023 (9)	−0.0039 (9)
C3	0.0422 (13)	0.0505 (14)	0.0305 (11)	−0.0031 (11)	−0.0043 (9)	−0.0084 (10)
C4	0.0393 (13)	0.0388 (13)	0.0467 (13)	−0.0048 (10)	−0.0005 (10)	−0.0125 (10)
C5	0.0418 (13)	0.0314 (11)	0.0402 (12)	0.0017 (10)	0.0045 (10)	0.0001 (9)
C6	0.0382 (12)	0.0387 (12)	0.0327 (11)	0.0021 (10)	−0.0036 (9)	−0.0026 (9)
C7	0.0326 (11)	0.0372 (12)	0.0322 (10)	0.0025 (9)	−0.0028 (9)	−0.0055 (9)
C8	0.0275 (11)	0.0361 (12)	0.0357 (11)	0.0019 (9)	−0.0004 (9)	−0.0001 (9)
C9	0.0495 (14)	0.0409 (13)	0.0344 (11)	−0.0059 (11)	−0.0005 (10)	0.0009 (10)
C10	0.0517 (15)	0.0451 (14)	0.0397 (12)	−0.0089 (11)	−0.0066 (11)	−0.0024 (10)
C11	0.0407 (13)	0.0364 (13)	0.0554 (14)	−0.0050 (10)	−0.0027 (11)	0.0008 (11)
C12	0.0501 (15)	0.0462 (15)	0.0492 (14)	−0.0058 (12)	0.0068 (11)	0.0128 (11)
C13	0.0425 (13)	0.0523 (15)	0.0370 (12)	−0.0029 (11)	−0.0035 (10)	0.0038 (10)

### Geometric parameters (Å, °)

**Table:**

Br1—C5	1.898 (2)	C5—C6	1.370 (3)
F1—C11	1.358 (3)	C6—H6	0.9300
O1—C2	1.350 (2)	C7—H7	0.9300
O1—H1	0.8176	C8—C9	1.385 (3)
N1—C7	1.274 (3)	C8—C13	1.387 (3)
N1—C8	1.423 (3)	C9—C10	1.381 (3)
C1—C6	1.395 (3)	C9—H9	0.9300
C1—C2	1.400 (3)	C10—C11	1.366 (3)
C1—C7	1.455 (3)	C10—H10	0.9300
C2—C3	1.382 (3)	C11—C12	1.359 (3)
C3—C4	1.375 (3)	C12—C13	1.384 (3)
C3—H3	0.9300	C12—H12	0.9300
C4—C5	1.381 (3)	C13—H13	0.9300
C4—H4	0.9300		
			
C2—O1—H1	110.1	N1—C7—H7	119.3
C7—N1—C8	121.51 (18)	C1—C7—H7	119.3
C6—C1—C2	118.99 (19)	C9—C8—C13	118.8 (2)
C6—C1—C7	119.02 (19)	C9—C8—N1	125.01 (19)
C2—C1—C7	121.99 (19)	C13—C8—N1	116.18 (18)
O1—C2—C3	118.68 (18)	C10—C9—C8	120.5 (2)
O1—C2—C1	121.60 (19)	C10—C9—H9	119.7
C3—C2—C1	119.7 (2)	C8—C9—H9	119.7
C4—C3—C2	120.78 (19)	C11—C10—C9	118.8 (2)
C4—C3—H3	119.6	C11—C10—H10	120.6
C2—C3—H3	119.6	C9—C10—H10	120.6
C3—C4—C5	119.4 (2)	F1—C11—C12	118.8 (2)
C3—C4—H4	120.3	F1—C11—C10	118.5 (2)
C5—C4—H4	120.3	C12—C11—C10	122.7 (2)
C6—C5—C4	120.9 (2)	C11—C12—C13	118.4 (2)
C6—C5—Br1	119.87 (16)	C11—C12—H12	120.8
C4—C5—Br1	119.20 (17)	C13—C12—H12	120.8
C5—C6—C1	120.14 (19)	C12—C13—C8	120.9 (2)
C5—C6—H6	119.9	C12—C13—H13	119.6
C1—C6—H6	119.9	C8—C13—H13	119.6
N1—C7—C1	121.38 (19)		
			
C6—C1—C2—O1	179.65 (18)	C6—C1—C7—N1	178.87 (18)
C7—C1—C2—O1	0.1 (3)	C2—C1—C7—N1	−1.6 (3)
C6—C1—C2—C3	−0.4 (3)	C7—N1—C8—C9	9.7 (3)
C7—C1—C2—C3	180.0 (2)	C7—N1—C8—C13	−171.9 (2)
O1—C2—C3—C4	−179.3 (2)	C13—C8—C9—C10	−0.4 (3)
C1—C2—C3—C4	0.8 (3)	N1—C8—C9—C10	178.1 (2)
C2—C3—C4—C5	−0.5 (3)	C8—C9—C10—C11	−0.2 (4)
C3—C4—C5—C6	−0.2 (3)	C9—C10—C11—F1	−179.0 (2)
C3—C4—C5—Br1	179.31 (17)	C9—C10—C11—C12	0.5 (4)
C4—C5—C6—C1	0.5 (3)	F1—C11—C12—C13	179.2 (2)
Br1—C5—C6—C1	−179.00 (15)	C10—C11—C12—C13	−0.2 (4)
C2—C1—C6—C5	−0.2 (3)	C11—C12—C13—C8	−0.3 (4)
C7—C1—C6—C5	179.4 (2)	C9—C8—C13—C12	0.6 (4)
C8—N1—C7—C1	−178.11 (18)	N1—C8—C13—C12	−177.9 (2)

### Hydrogen-bond geometry (Å, °)

**Table:**

*D*—H···*A*	*D*—H	H···*A*	*D*···*A*	*D*—H···*A*
O1—H1···N1	0.82	1.89	2.612 (2)	146

## References

[bb1] Allen, F. H., Kennard, O., Watson, D. G., Brammer, L., Orpen, A. G. & Taylor, R. (1987). *J. Chem. Soc. Perkin Trans. 2*, pp. S1–19.

[bb2] Bernstein, J., Davis, R. E., Shimoni, L. & Chang, N.-L. (1995). *Angew. Chem. Int. Ed. Engl.* **34**, 1555–1573.

[bb3] Bruker (2005). *APEX2*, *SAINT* and *SADABS* Bruker AXS Inc., Madison, Wisconsin, USA.

[bb4] Sheldrick, G. M. (2008). *Acta Cryst.* A**64**, 112–122. 10.1107/S0108767307043930 18156677

[bb5] Spek, A. L. (2009). *Acta Cryst.* D**65**, 148–155. 10.1107/S090744490804362X PMC2631630 19171970

